# Stages of Change in Dairy Intake among Older Adults: Application of the Transtheoretical Model

**DOI:** 10.3390/ijerph19031146

**Published:** 2022-01-20

**Authors:** Cheng-Fen Chang, Jiun-Yi Wang, Tien-Ho Kuo, Ying-Lien Lin, Shang-Yu Yang

**Affiliations:** 1 Department of Healthcare Administration, Asia University, Taichung 41354, Taiwan; cfchang222@gmail.com (C.-F.C.); jjwang@asia.edu.tw (J.-Y.W.); 2 Department of Nursing, Ching Kuo Institute of Management and Health, Keelung 203301, Taiwan; 3 Department of Medical Research, China Medical University Hospital, China Medical University, Taichung 404332, Taiwan; 4 Department of Leisure Management, Tungnan University, New Taipei City 222304, Taiwan; thkuo@mail.tnu.edu.tw; 5 Department of Industrial and Information Management, National Cheng Kung University, Tainan 701401, Taiwan; r38021019@gs.ncku.edu.tw

**Keywords:** transtheoretical model, stages of change (SOC), dairy product intake, older adults, self-efficacy

## Abstract

Adequate dairy product intake can reduce the risk of chronic disease, mortality, low quality of life, and healthcare expenditure. However, the insufficient consumption of dairy products is a serious issue in Eastern societies. To the authors’ knowledge, few studies have explored dairy intake among Taiwanese older adults, especially using the transtheoretical model. The study aims were to address the following unknowns: (i) the distribution of dairy product intake behavior on stages of change (SOC); (ii) differences in variables (intake knowledge (IK), intake cons (IC), intake pros (IP), and intake self-efficacy (ISE)) among SOCs; (iii) discriminative abilities of variables on SOCs; and (iv) predictive ability of variables (IK, IC, IP, and ISE) for dairy product intake behavior on SOC for older adults. An explorative cross-sectional study was conducted to collect data from northern Taiwan using a questionnaire. A total of 342 older adults were recruited. Data were analyzed using multivariate analysis of variance, discriminant analysis, and multiple linear regression. There was a significant difference between the variables and SOCs. There was a better discriminant among the five SOCs. Dairy product intake behaviors were significantly associated with knowledge and self-efficacy in the pre-action stage, and with cons, pros, and self-efficacy in the post-action stage. In conclusion, appropriate nutritional empowerment could benefit older adults by improving dairy intake among the different SOCs.

## 1. Introduction

The 2013–2016 National Nutrition and Health Survey [1] showed that 80–90% of the Taiwanese population ingested less than one serving of dairy products (e.g., milk, long-lasting milk, milk powder, yogurt, and cheese) per day. This was most serious among older adults, especially those over 75 years old (93.4%) [1]. Previous research mentioned that dairy product intake might be associated with a higher risk of prostate cancer [2] and Parkinson’s disease [3]. The long-term influence of lactose intolerance behaviors may contribute to a later risk of osteoporosis. Conversely, some studies reported that older adults enjoyed many benefits from dairy product intake, such as decreased mortality and chronic disease risk (such as stroke, hypertension, hip fracture, Alzheimer’s disease, sarcopenia, and cardiovascular disease mortality risk) [4,5,6,7,8,9,10,11,12,13]. Other studies have reported that enough dairy product intake might reduce colorectal cancer by 10%, metabolic syndrome by 13%, obesity by 16%, and osteoporosis risk by 39% [10,11,14,15,16]. Overall, the benefits of dairy product intake outweigh its harm to human health. Unfortunately, even though we understand the benefits of dairy products for older adults, the problem of insufficient intake remains among older adults [1].

Reviews indicated that chronic diseases caused by dietary habits have seriously threatened older adults’ health and quality of life [5,17]. Adequate dairy product intake can effectively reduce the risks of chronic diseases. The aim of dairy products is to provide a dietary source of multiple micronutrients, including high biological value protein, calcium, phosphorus, magnesium, potassium, zinc, selenium, vitamin A, riboflavin (vitamin B_2_), and vitamin B_12_ [7,10,18,19,20,21]. Therefore, it is urgent to encourage dairy product intake by older adults for increasing and improving their health status [22]. By changing the dairy intake behaviors of older adults, it might be possible to generate positive effects. The transtheoretical model stages of change (TTM SOC) have long been considered a useful intervention approach in lifestyle modification programs [23]. The transtheoretical model (TTM) focuses on participants’ behavior changes [24] and illustrates how participants move through five stages of behavior change. It has been widely applied to dietary and exercise health behaviors; in particular, dietary behavior studies have supported the use of TTM [24,25].

**Figure 1:**
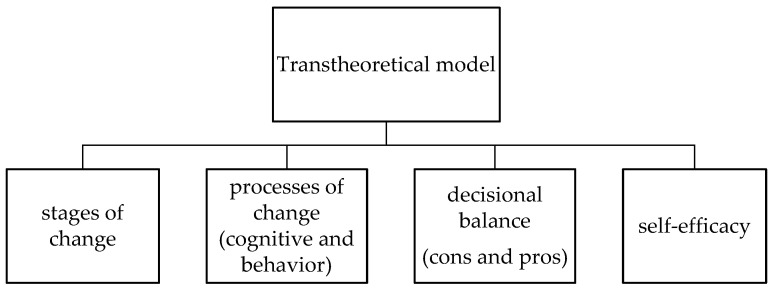
The four core constructs of TTM.

**Figure 2:**
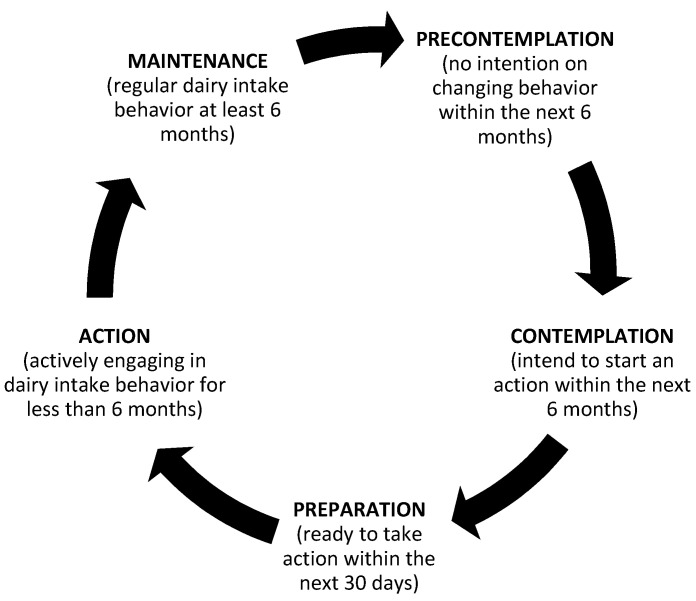
The stages of change in a transtheoretical model.

The TTM states that changing behavior is not a coincidence but a process, and different people have different SOC. Figure 1 is presented to illustrate the four core constructs of TTM. The four core constructs of TTM are: (i) SOC; (ii) processes of change; (iii) intake cons and intake pros (IC and IP); and (iv) intake self-efficacy (ISE) [26]. Healthy behavior change involves progressing through the following five SOC (first core construct of TTM): (i) pre-contemplation: no intention to take action within the next six months; (ii) contemplation: intending to start an action within the next six months; (iii) preparation: ready to take action within the next 30 days; (iv) action: actively engaging in dietary behavior for less than six months; and (v) maintenance: regular dietary behavior for at least six months [25,27] (Figure 2). Moving from earlier to later stages was dependent on the other three constructs. Moreover, the process of change included the second-higher order model (e.g., cognitive and behavioral processes), which have been used to revise experience and improve behavior [25]. Regarding the decisional balance, the pros and cons are the positive and negative aspects of modifying participants’ behavior [28]. The cons outweigh the pros in the earlier pre-contemplation stage of change [25]. Self-efficacy was persons’ belief in their own ability to execute the course of action required to achieve given goals [29]. The higher the SE involved in tasks, the more positive the emotions elicited from the tasks [29]. Therefore, higher SE could improve dietary behavior.

Many studies have demonstrated a significant association between the TTM and dietary intake behaviors in children or adolescents. However, there are few studies on the application of TTM in changing dairy intake behaviors for older adults. Therefore, the study aims were to answer the following four questions:

1. What is the distribution and frequency of dairy product intake behavior on SOC for older adults?
2. Are there any differences in variables (IK, IC, IP, and ISE) among the SOC for older adults?
3. Can the SOC of dairy product intake be effectively discriminated by variables (IK, IC, IP, and ISE) for older adults?
4. Can the dairy product intake behavior be predicted by variables (IK, IC, IP, and ISE) on the SOC for older adults?

Once the results were obtained, dietitians could use them to design or strengthen health strategies for older adults on different stages of dairy product intake. Moreover, it might be applied to reduce the burden of caregivers and the healthcare system.

## 2. Materials and Methods

### 2.1. Study Design and Recruitment

An exploration cross-sectional study was conducted between August and October 2020 in Zhongzheng District, Keelung City. Data were collected using a structured questionnaire. Interviewers were trained before visiting the participants, and they explained the purpose of the study to the participants. Written informed consent was obtained from all participants before enrollment. 

The inclusion criteria for the study were as follows: (i) aged ≥ 65 years and (ii) able to communicate in Mandarin Chinese or Taiwanese. If participants did not have sufficient cognitive or daily living functioning, they would lack the relevant knowledge and behavioral ability to purchase or consume dairy products. Therefore, we formulated relevant exclusion conditions. The exclusion criteria were as follows: (i) a Short Portable Mental State Questionnaire score of ≥ 3 and (ii) a Barthel Index score of < 91. The population in our research consisted of 9271 residents aged ≥ 65 years in Zhongzheng District, Keelung City, Taiwan [30]. Of these, 348 older adults were conveniently sampled from 26 administration units in Zhongzheng District; among them, six were excluded due to the questionnaire being incomplete. Consequently, a total of 342 participants were included in the study (98.3%). To test if the participants represent the population, the present study conducted χ^2^ testing on the age and gender of the participants and found no significant difference between the participants (age χ^2^ = 3.52, *p* = 0.172; gender χ^2^ = 2.30, *p* = 0.130) and the Zhongzheng District population. Therefore, the results indicated that the participants could effectively represent the population. The study was approved by the Ethics Committee of the Ministry of Health and Welfare at Taipei Hospital (registration ID No.TH-IRB-0021-0018).

### 2.2. Questionnaire

The structured questionnaire comprised five sections that were designed based on previous studies [31] in order to analyze the beliefs regarding IK, IC, IP, and ISE of dairy product consumers. To ensure validity, a public health expert, a dietitian, and a health education expert were invited to give feedback. The content validity index of all sections was verified to have satisfactory validity (>0.8). 

Section 1 consisted of background data, including gender, age, education, and chewing ability.

Section 2 was composed of an IK scale (four items) (such as eating enough dairy products every day increases calcium and prevents osteoporosis) to measure the benefit and nutritional knowledge of dairy product intake. The scale was rated on a two-point scale, scored as 1 = agree and 0 = disagree/neutral; the lowest total score was 0 and the highest was 4. A high total score for the scale indicated a high capacity for IK. The Cronbach’s alpha value for the IK scale was 0.82 (Table 1).

Section 3 featured the IC and IP of dairy product intake to test the obstacles and positive results of dairy product intake. The scale was composed of two subscales: IC (7 items), with items such as “I do not like the taste of dairy, so I do not drink it,” and IP (4 items), with items such as “Drinking or eating dairy products can effectively slow weakness or maintain muscle mass.” The scale was rated on a 5-point Likert scale, ranging from 1 = never to 5 = always; the lowest scores were 7 and 4, and the highest scores were 35 and 20, respectively. A high total score for the scale (or the respective subscales) indicated a high capacity for IC and IP. The Cronbach’s alpha values for the IC and IP scales were 0.96 each (Table 1).

Section 4 was an ISE scale on dairy product intake, which focused on overcoming difficulties in special circumstances and performing dairy product intake behavior. This scale was comprised of nine items. An example item is “To eat enough dairy products every day, I....” The scale was rated on a 5-point Likert scale ranging from 1 = very difficult to 5 = very easy; the lowest total score was 9 and the highest was 45. A high total score on the scale indicated a high capacity for ISE. The Cronbach’s alpha value for the ISE scale was 0.96 (Table 1).

Section 5 covered SOC of dairy product intake (1 item) and dairy product intake behavior (1 item). An example item is “I would like to consider the dairy product intake stage and behavior.” The SOC contained precontemplation = 1: no intention to take action within the next six months; contemplation = 2: intend to start an action within the next six months; preparation = 3: ready to take action within the next 30 days; action = 4: actively engaging in dietary behavior for less than six months; and maintenance = 5: regular dietary behavior for at least six months [25,27]. The dairy intake behavior tested the intake frequency of 1.5–2 servings per day/week within a month: one day = 1, two days = 2 … and so on. A high total score on the scale indicated good dairy product intake behaviors. To avoid making a mistake in the assessment of dairy product exchange, the interviewer would use pictures to illustrate the categories (Table 1).

### 2.3. Statistical Analysis

All statistical analyses were performed by IBM SPSS Statistics v.27. Statistical significance was set at *p* < 0.05. Quantitative variables were expressed as mean±SD. Descriptive statistics were used to show the distributions of demographic characteristics and other studied variables. The analysis also compared averages of four variables (IK, IC, IP, and ISE) in the SOC using multiple analysis of variance (MANOVA). Scheffe’s test was used for post-hoc analyses. Discriminant analyses were used to test the discriminant power of variables (IK, IC, IP, and ISE) on the SOC of dairy product intake. After the adjustment of the demographic variables (age, sex, educational level, and chewing ability), a multiple regression model was performed to assess the correlations between dairy product intake and the four variables on the SOC. In addition, each multiple regression model was performed by collinear diagnosis. In the multiple regression model, the variance inflation factors of all independent variables were checked as suggested [32] and no remarkable collinearity was observed.

## 3. Results

### 3.1. Baseline Characteristics of the Participants and Dairy Product Intake

The participants’ gender, age, education, chewing ability, and dairy product intake behavior are presented in Table 2. Out of the 342 participants, 286 participants (83.6%) were in the pre-action stage (distributed predominantly across precontemplation, contemplation, and preparation). Meanwhile, 16.7% of males and 31.9% of females were in the preparation stage. The mean age of participants was 76.6 ± 8.7 years, with females comprising 68.8% of total participants. Over half of the participants were literate. More than half of the participants had normal chewing ability. Almost all of the participants (93.3%) failed to reach the recommended daily intake of dairy products. On average, the participants took 1.6 (±2.0) days to reach the recommended daily allowance of dairy products (1.5–2 servings/day). The majority of participants on the education, chewing ability variables were in the preparation stage. Around half of the participants’ dairy product intake behavior was in the preparation stage. 

### 3.2. Differences in Variables (IK, IC, IP, and ISE) among the SOC 

To understand the differences among older adults in the SOC of dairy product intake, MANOVA was used, and the results are shown in Table 3. The MANOVA revealed a significant difference in IK, IC, IP, and ISE among the five stages (IK F = 6.25, *p* < 0.001; IC F = 3.07, *p* < 0.05; IP F =18.04, *p* < 0.001; ISE F = 36.39, *p* < 0.001). After post hoc analysis, all pairwise comparisons indicated the following. In the IK part, there was a significant difference; the means of contemplation, preparation, action, and maintenance were higher than in the precontemplation stage. In the IP part, the means of contemplation, preparation, action, and maintenance were higher than in the precontemplation stage; the means of action and maintenance were higher than in the precontemplation stage. Furthermore, the mean of maintenance was higher than in the preparation stage. In the ISE part, the means of contemplation, preparation, action, and maintenance were higher than in the precontemplation stage; the means of preparation, action, and maintenance were higher than in the contemplation stage. Moreover, the means of action and maintenance were higher than in the preparation stage. Finally, in the IC part, none of the pairwise comparisons showed differences.

### 3.3. Discriminant Analysis of Variables on Stages of Change (SOC)

To understand if the relevant variables can effectively discriminate the SOC in dairy product intake among older adults, a MANOVA was conducted. It showed that the differences in the degree of IK, IC, IP, and ISE for older adults in different SOC had a Wilks’ Lambda of 0.62, reaching a statistically significant level (F = 10.93, *p* = 0.00). Table 4 shows the discriminative results of IK, IC, IP, and ISE in different SOC; four typical functions were chosen, and the eigenvalues values were 0.54, 0.054, 0.02, and 0.00, respectively, while the Wilks’ Lambda were 0.62, 0.95, 0.98, and 1, respectively (χ^2^ = 162.48, df = 16; χ^2^ = 17.70, df = 9; χ^2^ = 5.61, df = 4; χ^2^ = 0.001, df = 1). The first and second typical discriminative functions showed the significantly discriminative effect. The ISE (0.82) of the first function and the second function of IC (1.00) had higher relative importance and higher discriminative power than the other predictive variables between the different SOC. Regarding the model prediction, it was acceptable, because 49.4% of the SOC of dairy intake among older adults could be correctly classified by IK, IC, IP, and ISE (100% is excellent). Based on the Press Q score, the discriminant analyses were outstanding, because the score was 73.46 > 6.63 [33].

### 3.4. The Association between Variables and Dairy Product Intake on SOC

The results of the multiple regression analysis are shown in Table 5. After controlling the demographic variables (age, gender, education level, and chewing ability), the results indicated that dairy product intake behavior had a significant association with IK, IC, IP, and ISE among SOC, except for in the maintenance stage. In the precontemplation stage, IK (B = 0.60, *p* = 0.02) and ISE (B = 0.47, *p* = 0.00) were significant factors with intake behavior. In the contemplation stage, ISE (B = 0.32, *p* = 0.00) was a significant factor. In the preparation stage, IC, IP, and ISE (B = 0.21, *p* = 0.00; B = 0.16, *p* = 0.03; B = 0.18, *p* = 0.01) were significant factors. In the action stage, ISE (B = 0.18, *p* = 0.04) was a significant factor. However, in the maintenance stage, there were no significant factors with intake behavior.

## 4. Discussion

The present study indicated that the significant differences of IK, IC, IP, and ISE and the good discriminant among SOC would provide community and clinical settings with the methods to increase dairy product intake among all older adults. In addition, SE had a significant association with dairy product intake. Therefore, it is important to improve older adults’ dairy intake of SOC by improving SE.

### 4.1. Description of Demographic Characteristics and Dairy Product Intake Behavior

The results of the present study showed that the dairy product intake of most participants (93.3%) is similar to the results of a previous study in which 73% of older adults did not consume enough dairy [34]. Moreover, 83.7% of participants were in the pre-action stage (distributed predominantly across precontemplation, contemplation, and preparation), and 16.3% were in the post-action stage (distributed predominantly across action and maintenance). Therefore, it is suggested that we should educate older adults about IK in the pre-action stage to promote their health in the future. 

In this study, only 6.7% of participants consumed 1.5–2 servings of dairy products per day, however, the majority (93.3%) did not reach the nationally recommended allowance. Similarly, the results of the National Dietary Survey (NAHSIT 2013–2016), pointed out that 80–90% of the population consumed less than one serving of dairy products per day. The worst situation was that 93.4% of older adults (≥75 years) consumed less than one serving of dairy products per day [1]. Compared to the United States and Europe, a plausible reason for the low intake of dairy products in Asia is that milk is not as popular in Taiwanese society. In particular, eating rice as a dietary staple contributes to malnutrition and protein deficiency in older adults in Taiwan. The other main reason was the high prevalence of lactose intolerance in Taiwan [35]. As result, older adults with malnutrition increased the risk of having chronic disease (e.g., sarcopenia) due to insufficient protein intake [36,37].

### 4.2. Different in Variables between the SOC 

The findings of the present study showed a significant discrepancy among different SOC of dairy product intake for IK, IC, IP, and ISE (Table 3). A TTM meta-analysis [38] found that participants at the precontemplation stage were significantly less likely to meet health-related goals than those in later stages. Similarly, the present study mentioned the lower mean of dairy product intake among IK, IC, IP, and ISE for older adults at the precontemplation stage, in comparison with the action and maintenance stages, providing further support on the validity of the SOC of the TTM. Meanwhile, a lack of dairy product intake was found to be caused by insufficient knowledge and inappropriate dietary habits among Taiwanese older adults [1]. Asian countries exhibit a much lower demand for dairy products than Western countries. In addition, older adults often consume fewer dairy products on account of lactose intolerance, the smell of milk, or even serious dairy allergies [8]. In each stage, the mean ± SD of the IC variable of older adults were similar, leading to a decline correlation between the variables. To date, the reasons are not yet fully understood and should be further investigated and discussed in the future. 

Meanwhile, IP was also an important variable for the dairy product intake of older adults (Table 3). This study showed a higher score in the post-stage than in the pre-stage for both IP and SE. In contrast, the study showed a lower mean score in the post-stage than in the pre-stage for IC. Some chronic diseases might be prevented if older adults consume just one serving of dairy product daily [2,15]. Therefore, based on the results of this study, it is suggested that we should promote health education to emphasize the advantage of dairy product intake in the pre-stage and encourage dairy product intake in the post-stage for older adults in the future. Even the strategies of national public health education could be focused on encouraging older adults to reach one daily serving of dairy in the pre-stage and then move toward the next stage until they reach the maintenance stage. 

### 4.3. Discriminant of Variables on Stages of Change (SOCs) 

The present study indicated that the SOC can be applied to the dairy product intake behavior of older adults, for which its related variables can effectively distinguish the SOC of dairy product intake among older adults (Table 4). It is helpful for dieticians to design health courses at different stages for the prevention of frailty and sarcopenia occurring in older adults. The study results were similar to previous studies [39]; although, the relevant researches are still very limited and further studies are still needed.

### 4.4. The Association between Variables and Dairy Product Intake on SOC

The present study showed a positive correlation between IK and dairy intake behavior in the pre-contemplation stage (*p* = 0.02) (Table 5). These findings were consistent with results conducted by Park et al., whose study involving 199 community-dwelling older adults. The results showed that intake knowledge could strengthen older adults’ calcium supplementation and vitamin D intake [40]. This implies that in the pre-contemplation stage, older adults need more intake knowledge to enhance dairy product intake behaviors. 

The IC and IP were significantly correlated with dairy product intake in the preparation stage, but not in the other SOC (Table 5). In the preparation stage, IC had a positive correlation with dietary intake behaviors. Older adults might be scared that their health will become worse, so that the increase of the IC score led to more frequency of dairy intake behaviors [2,3,4,5,6,7,8,9,10,11,12,13]. Furthermore, IP made an impact on dairy product intake merely in the preparation stage (Table 5). In fact, dairy intake was not as popular in Taiwan as in Western societies [35]. Therefore, based on the finding of this study, policymakers would be able to design strategies that enhanced more the IPs (advantages) and ICs (disadvantages) of dairy product intake to improve dairy intake behavior at this stage. Moreover, promoting older adults’ dairy product intake behaviors rapidly moves on to the next stage (action stage). 

Moreover, this study showed a positive relation between ISE and dairy intake behavior in the pre-contemplation stage (Table 5). The results of the study were similar to those from the study conducted by Park et al., who pointed out that self-efficacy could intensify the calcium supplementation and vitamin D intake behaviors of older adults [40]; a study carried out by Hsu et al. who conducted a quasi-experimental study design mentioned 68 older Asian women in a community changed their dairy intake behavior and improved their dairy intake self-efficacy through empowerment education [41]. Consequently, ISE was the best predictor of dairy product intake. Older adults would have more positive expectations and dairy product intake behaviors along with higher ISE. 

The results of the study showed IK, SE, IC, IP, and dairy intake behavior differed significantly by stage. Hence, a health education strategy for older adults’ dairy intake behaviors can be designed based on the SOC. Furthermore, the prevention of low dairy intake behaviors among older adults could lie in social support, health promotion, and nutrition education in communities. Suggestions for policymakers and practitioners, like dieticians, are as follows. On the one hand, to increase older adults’ intention for more dairy product intake, policymakers could combine the government budget and additional manpower to popularize nutrition empowerment education through clinics and community. The government could even provide dairy discount coupons to older adults to promote the consumption of dairy products. On the other hand, dieticians might design courses or workshops which transmit knowledge on dairy product intake in accordance with individual’s stage of dairy product intake. By holding such activities as sharing food or DIY cooking, dieticians could effectively improve older adults’ intention to consume dairy products. 

### 4.5. Limitations

There are several limitations in the present study. First of all, the study with a cross-sectional research design failed to explain the causations between dairy product intake and processes of change (knowledge), decisional balance (cons and pros), and SE. The causes of dairy product intake are recommended in future studies. Second, this study has limited generalizability and cannot represent the entire population. So, future studies could include a variety of populations in different cities. In addition, examining older adults grouped by adding other variables to discover more influencing factors at various stages will help to create an effective behavioral change strategy. Meanwhile, age stratification would be suggested and considered in future researches. Third, it might contain a bias in all questionnaires conducted by participants’ self-reported information and social expectations. Last, some data, such as anthropometrical measurements, overall health status, and the lactose tolerance ability, could not be collected because of an incapacity of concentration over a long period of time for older adults. Nevertheless, the above items could be taken into consideration in future studies. 

## 5. Conclusions 

There were significant differences between the variables (IK, IC, IP, and ISE) and the five SOCs. There was a better discriminant among the five SOCs. TTM constructs, including processes of change (knowledge), IC, IP (intake cons and pros), and ISE were significantly correlated with dairy product intake. More attention should be paid to dairy product intake behaviors in the pre-action stage for older adults. Moreover, in the pre-action stage, dietitians should promote nutrition empowerment (e.g., health education promotion and dairy product DIY cooking activities) in community or clinical settings to improve dairy product intake behaviors and prevent related chronic diseases in older adults. 

## Figures and Tables

**Table 1:** Characteristics of questionnaires.

Basic Theoretical	Name of Scale	No. of Items	Aim of Questionnaire
TTM-processes of change	IK	4	to measure the benefit and nutrition knowledge
TTM-decisional balance	IC	7	to test the obstacles results
TTM-decisional balance	IP	4	to test the positive results
TTM-self-efficacy	ISE	9	to focus on overcoming difficulties in special circumstances and performing dairy product intake behavior
TTM-stages of change	SOC	1	to assess the SOC of dairy product intake

IK: intake knowledge, IC: intake cons, IP: intake pros, SOC: stages of change, ISE: intake self-efficacy (refer to Prochaska et al., 1994).

**Table 2:** Baseline characteristics of the study population, stratified by stages of change (n=342).

Characteristics	All (n = 342)	Pre- Contemplation	Con- Templation	Pre- Paration	Action	Maintenance
Gender						
male, n (%)	107 (31.2)	12 (3.5)	20 (5.8)	57 (16.7)	11 (3.2)	7 (2.0)
female, n (%)	235 (68.8)	30 (8.8)	58 (17)	109 (31.9)	22 (6.4)	16 (4.7)
Age(years), n (%)	342 (100)	42 (12.3)	78 (22.8)	166 (48.5)	33 (9.7)	23 (6.7)
M ± SD	76.6 ± 8.7	80.4 ± 8.4	77.8 ± 8.2	73.9 ± 8.2	78.8 ± 9.2	81.7 ± 8.7
65–74, n (%)	156 (45.6)	17 (5)	22 (6.4)	99 (28.9)	10 (2.9)	8 (2.3)
75–84, n (%)	109 (31.9)	14 (4.1)	33 (9.6)	46 (13.5)	11 (3.2)	5 (1.5)
≥85, n (%)	77 (22.5)	11 (3.2)	23 (6.7)	21 (6.1)	12 (3.5)	10 (2.9)
Education						
literacy, n (%)	261 (76.4)	30 (8.8)	59 (17.3)	132 (38.6)	23 (6.7)	17 (5.0)
illiteracy, n (%)	81 (23.7)	12 (3.5)	19 (5.6)	34 (9.9)	10 (2.9)	6 (1.8)
Chewing ability						
normal, n (%)	289 (84.4)	36 (10.5)	67 (19.6)	143 (41.8)	22 (6.4)	21 (6.1)
difficulty, n (%)	53 (14.5)	6 (1.8)	11 (2.2)	23 (6.7)	11 (3.2)	2 (0.6)

**Table 3:** MANOVA analysis between variables and stages of change (n = 342).

	N (%)	M ± SD	F	Scheffe’s
Intake knowledge			6.25 **	e, d, b, c > a
a. precontemplation	42 (12.3)	0.25 ± 0.40		
b. contemplation	78 (22.8)	0.54 ± 0.41		
c. preparation	166 (48.5)	0.48 ± 0.38		
d. action	33 (9.7)	0.61 ± 0.42		
e. maintenance	23 (6.7)	0.66 ± 0.40		
Intake cons			3.07 *	
a. precontemplation	42 (12.3)	2.29 ± 1.37		
b. contemplation	78 (22.8)	2.57 ± 0.83		
c. preparation	166 (48.5)	2.58 ± 0.65		
d. action	33 (9.7)	2.36 ± 0.84		
e. maintenance	23 (6.7)	2.05 ± 0.73		
Intake pros			18.04 **	e, d > b, a; e > c; c, b > a
a. precontemplation	42 (12.3)	2.47 ± 1.05		
b. contemplation	78 (22.8)	3.10 ± 0.75		
c. preparation	166 (48.5)	3.22 ± 0.55		
d. action	33 (9.7)	3.63 ± 0.81		
e. maintenance	23 (6.7)	3.78 ± 0.84		
Intake self-efficacy			36.39 **	e, d, c > b > a; e > c
a. precontemplation	42 (12.3)	1.80 ± 0.81		
b. contemplation	78 (22.8)	2.52 ± 0.69		
c. preparation	166 (48.5)	2.81 ± 0.52		
d. action	33 (9.7)	3.34 ± 0.91		
e. maintenance	23 (6.7)	3.50 ± 0.94		

* *p* < 0.05, ** *p* < 0.01.

**Table 4:** Discriminant analysis of variables on stages of change (n = 342).

	Standardized Canonical Discriminant Function Coefficients				Structure Coefficients			
	1st Function	2nd Function	3rd Function	4th Function	1st Function	2nd Function	3rd Function	4th Function
Intake knowledge	−0.02	0.10	1.10	−0.45	0.33	0.10	1.00 *	0.08
Intake cons	0.17	1.00	−0.17	0.04	−0.02	1.00 *	−0.05	0.09
Intake pros	0.44	−0.11	−0.24	1.11	0.63	−0.08	0.31	0.71 *
Intake self-efficacy	0.82	0.06	−0.25	−0.61	0.90 *	−0.16	−0.13	−0.40
eigenvalue	0.54	0.04	0.02	0.00				
Wilks’λ	0.62	0.95	0.98	1				
49.4% of the original group observations were correctly classified. Press’ Q = 73.46								

* *p* < 0.05.

**Table 5:** Multiple regression analysis of dairy product intake behavior among stages of change (n = 342).

	Dairy Product Intake Behavior														
	Precontemplation			Contemplation			Preparation			Action			Maintenance		
Independent Variable	B	β	*p*	B	β	*p*	B	β	*p*	B	β	*p*	B	β	*p*
Intake knowledge	0.60	0.36	0.02 *	0.22	0.15	0.11	0.13	0.12	0.20	0.37	0.39	0.17	0.05	0.05	0.90
Intake cons	0.01	0.02	0.88	0.05	0.06	0.42	0.21	0.35	0.00 ***	0.04	0.09	0.64	0.01	0.02	0.94
Intake pros	0.06	0.10	0.55	0.02	0.02	0.84	0.16	0.22	0.03 *	0.17	0.35	0.22	0.15	0.28	0.36
Intake self-efficacy	0.47	0.55	0.00 ***	0.32	0.37	0.00 ***	0.18	0.24	0.01 **	0.18	0.42	0.04 *	0.08	0.16	0.62
R^2^	0.61			0.63			0.21			0.49			0.50		
Adj R^2^	0.49			0.58			0.16			0.29			0.15		
F	5.30 ***			12.82 ***			4.55 ***			2.42 *			1.44		

* *p* < 0.05; ** *p* < 0.01; *** *p* < 0.001.

## Data Availability

The datasets generated during and analyzed during this study are available from the corresponding author on reasonable request.
